# Parent Perspectives: Part 1—Considerations for Changing the NICU Culture †

**DOI:** 10.3390/children10111735

**Published:** 2023-10-26

**Authors:** Jessica N. DiBari, LaToshia Rouse

**Affiliations:** 1 U.S. Department of Health and Human Services, Health Resources and Services Administration, Maternal and Child Health Bureau, Rockville, MD 20857, USA; 2 Certified Doula at Birth Sisters Doula Services and Patient Engagement Consultant, Knightdale, NC 27545, USA; contact@birthsistersdoula.com

**Keywords:** neonatal intensive care unit, preterm birth, life course, collaborative care models

## Abstract

While publications that aim to reflect the parent perspective are increasingly common in the medical literature, few are authored by parents in their own words. As mothers with lived and professional experience in the Neonatal Intensive Care Unit (NICU), we believe this perspective is vital to improving health outcomes. We are writing from a life course health development framework that regards health as an active process that develops over time with the integration of physical, social, emotional, and relational components. Health development trajectories are shaped by the family and community ecosystems that surround each child. This means that the child’s ability to thrive is strongly linked to the family’s health and well-being. These links are not being given sufficient attention in clinical practice. Psychological distress, trauma, and grief are common family experiences in the NICU. Left unaddressed, they can negatively impact parent-child bonding. Drawing on life course principles, we make a series of recommendations for changes to practice to enable NICUs to better serve children and families, and better prepare families for the post-NICU experience. These include setting a positive tone in the NICU, creating a nurturing, personalized environment; addressing the social determinants of health; supporting families to develop a ‘growth’ mindset; and communicating in an optimistic, positive manner. Building trust is key to ensuring families feel supported and can be promoted through establishing equitable collaborative models of care. Peer support, doulas, and community health worker engagement can facilitate early interactions crucial to the child’s developmental progress and family healing.

## 1. Introduction

The experience of a Neonatal Intensive Care Unit (NICU) family is starkly different from a family that has given birth to a healthy newborn. Joy and celebration are replaced with fear and trepidation. The fragile life of a NICU baby is at stake and families must cope with an uncertain future. Often there are risks communicated during pregnancy that will increase anxiety not knowing what is to come post birth. This fear of the unknown has an impact on all parents and coping strategies are highly varied at the individual level.

While publications based on parent focus groups and interviews are increasingly common in the medical literature, few are authored by parents in their own words, or reflect the parents’ own integrated perspective on what steps might improve care. This perspective is vital to truly co-develop interventions that are likely to improve health outcomes across the life course.

This paper is written from a life course health development framework, which provides a comprehensive conceptual model that regards the development of health as an active process that occurs over time and integrates physical, social, emotional, and relational components. Health development trajectories are shaped by the family and community ecosystems that surround each child [1]. It acknowledges the importance of the links between parental and family health and well-being and the child’s ability to thrive. These links are not being given sufficient attention in clinical practice. The Health Resources and Services Administration, Maternal and Child Health Bureau recently introduced the Blueprint for Change to provide a framework centered around four critical areas: health equity, family and child well-being and quality of life, access to services, and financing of services [2]. Consistent with these priorities, recent work by the LCIRN has identified twelve characteristics that are important to consider when planning life course interventions that are designed to improve health trajectories [3]. Life course interventions (1) aim to optimize health trajectories; are (2) developmentally focused; (3) longitudinally focused; (4) strategically timed; (5) designed to address multiple levels of the ecosystem where children are born, live, learn, and grow; and (6) vertically, horizontally and longitudinally integrated to produce a seamless, forward tilting, health optimizing system. Interventions (7) support emerging health development capabilities; (8) are collaboratively co-designed by transdisciplinary research teams including stakeholders; and incorporate (9) family-centered; (10) strengths-based; and (11) anti-racist approaches; with a (12) focus on health equity that incorporates anti-bias and trauma-informed care. In this paper, we draw on our experience as NICU parents to apply this framework to the care of families and children in the NICU. Finally, we draw conclusions and make recommendations for changes to practice that could enable NICUs to better serve children and families in ways that are consistent with life course principles.

## 2. Psychological Distress and Early Bonding/Attachment

Providing families with support as early as pregnancy is necessary to better prepare for the unexpected and the twists and turns of a NICU journey. Like sensitive periods in human development when specific experiences can have an outsized effect on future functioning, there are pivotal moments in the NICU journey that are not just fleeting moments in time but traumatic events that have a lasting effect. The NICU is a new land with a new language for families. No words can describe the feeling of the NICU “SWAT” team rushing in to resuscitate your child, the piercing sounds of the monitors every time the heart rate and oxygen levels fall to dangerously low levels, or seeing your child turn blue and lifeless. It is paralyzing. Feelings of remorse, regret, blame, and trying to grapple with the why/how can be all-consuming. Sometimes there is an overwhelming feeling of your body failing you. Families entering the NICU usually come into the space with some amount of trauma associated with the events leading up to the NICU stay. The NICU is also very much a place for grieving parents. The grief could be related to the timing of the delivery, the experience during pregnancy, the circumstances of the delivery, or the health of the baby. This grief is often layered upon what families may be already coping with, including physical, sexual, and emotional abuse, childhood neglect, mental health issues, substance abuse, poverty, racism, discrimination, and/or oppression. The pregnancy experience is processed during the NICU stay and beyond. Regardless of the length of time in the NICU, families struggle with NICU admission. The demands of a NICU stay are not quantifiable. The emotional toll on families is too difficult to put into words and even many years post NICU they still linger [4]. Families need support to emotionally navigate the NICU journey.

The support team can help families cope if their baby was born preterm and help the family accept this outcome. Every parent’s first thought is what did I do wrong? The healthcare team can help the family process the fact that the baby requires NICU intervention and understand that they may never know why their baby was born early. It is also important to accept the fact that suboptimal intrauterine conditions led to the need for early birth. When the baby is in utero so much is unknown about the condition of the environment and very little can be done in terms of interventions during pregnancy. In the NICU, the careful monitoring of every aspect of the baby provides care that is incredibly precise and tailored to meet the needs of the developing baby. Mothers may feel a sense of relief after their baby is born that the outcome is now completely out of their control. Others may have an emotional response to this lack of control. Providers can help families realize that a preterm outcome, NICU intervention, and continued growth outside of the womb are what is best for the developing baby.

## 3. Bonding with a NICU Baby

Families may be hesitant to interact with a fragile NICU baby at first. It is terrifying to see your child in an incubator with dozens of wires and monitors. The list of concerns is extensive such as brain bleeds, infections, collapsed lungs, blood transfusions, and feeding intolerances. Seeing your child stop breathing and requiring intervention to stay alive in and of itself is hard to watch. The hours are long and the mental strain it has on the family is great. Providers and nurses can help combat feelings of helplessness by encouraging family and child bonding and helping the family to see how the child has their own distinctive personality even early on. *Peer support, doulas and community health worker engagement can also help families feel more comfortable interacting with their baby.*

Fostering opportunities for families to connect with their babies is critically important. In those early weeks, gentle touches encourage bonding and a connection between family members and the child [5]. Positive interactions can stimulate brain connectivity and support brain development in babies while providing healing opportunities for the parents of the NICU baby. Babies, regardless of their health status, benefit from their parent’s touch. The medical team can encourage families to hold their child’s hand, speak and sing to him/her, koala care or skin-to-skin when they are ready, and what some NICU’s call hand hugs where you reach through the holes in the incubator and gently place a hand on top of the head and feet as if you were measuring the baby’s length. Hand hugs are a great entry point to build confidence and connection. From Jessica’s experience: Looking back at photos, I can see that my son was aware of our presence. The first time we held our son in the NICU we were scared and needed encouragement. He was so fragile and unstable. *These early interactions are critical to the child’s progress, growth, and development as well as family healing.*

Several months into our NICU stay, my other son, age 4 at the time, was allowed to visit. A lifelong bond was established through hand holds, forehead kisses, and wagon rides dragging medical equipment through the NICU halls. One of the hospitals gave the visiting son a sticker that said “big bro” every time he passed through security to visit. This may seem insignificant, but he wore this sticker like a badge of honor and even decorated our house with them. *This may have not been a typical newborn experience, but I am forever grateful for the precious moments that highlight the strength and support of our family, friends, and care team.*

One of the nurses insisted that we take a photo with my husband’s wedding band around my son’s arm in the first few days of life. We were resistant. We did not want to cause him any distress. This image is a constant reminder of how far we have come. After one week of life the wedding band was too small to fit around my son’s tiny arm. *Reflecting on accomplishments and progress gives hope for the future.*

## 4. Setting the Tone in the NICU

One day, we arrived at the NICU and saw on the entryway monitor a status update next to a baby’s name. The screen read “deceased”. The words cut through our hearts and, even though it was not our child, we mourned. Some hospitals use the symbol of a purple butterfly when a family has experienced the loss of a child. This can be placed outside the door of the room with no explanation.

Optimism, positive energy, and kindness can really have an impact on a family’s NICU experience. A simple smile, hug, or an encouraging comment can help families stay positive. An ounce of positivity breeds hope! At one point, my son was undergoing a routine brain scan to look for brain bleeds and even though he was just over a pound he was punching and swatting at the technician. He was fighting! Rather than focusing on how tiny and fragile he was and the fear of the results of the brain scan, we shifted the conversation to how feisty and resilient he was. Everyone in the room was laughing and smiling. *Many families have stories of how they were able to see their baby’s personality at different stages of development while in the NICU. These positive associations help reduce the trauma associated with being in the NICU.*

Furthermore, engagement with families can improve morale and create a positive atmosphere. Setting expectations, including the expected duration of the NICU stay, helps families to plan. Some teams have created milestone cards and incorporated anticipatory guidance as a regular part of care. The way we communicate risks and eminent threats influences one’s mindset [6]. Focusing on the present and any warning signs shifts attention to actionable next steps such as screening and increased monitoring rather than future risks that may or may not present themselves. *The risk of serious diagnoses that will persist throughout life is not taken lightly and should not be discussed as a potential threat unless there are early signs and/or symptoms that will likely lead to this diagnosis.*

## 5. Creating a Nurturing Environment

The NICU can be an uninviting environment with a very sterile feel. The care team can encourage families to create a warm nursery-like environment to provide stimulation, including a mobile, black and white images, and photos displayed of the family around the room. Even a growth chart can be displayed to track the child’s progress. Encourage the family to speak to the baby, sing to the baby, and read books. Treat the baby like a newborn to the extent possible! These coping strategies help improve morale and support the baby’s development. Some NICUs even hire musicians who visit each room 1–2x per week. *There is an evidence base for music as therapy [7]. A study of 1092 neonates “showed music therapy had a significant influence on preterm infant’s heart rate, respiratory rate, oral feeding volume, stress level, and maternal anxiety” [8]. It also breaks up the day and changes the mood in the NICU for families.*

## 6. Supporting, Collaborating, and Communicating with Families

We need to build a system of care that addresses the social determinants of health [9]. Support comes in many shapes and forms. Doctors, nurses, social workers, and other professionals and peers can help families develop a growth mindset. What is not possible today may be possible tomorrow. Staff using words like “yet” is supportive language. Hearing clinical staff say your baby has not done this “yet” leaves room for hope that they will in the future. Parents of typically developing children often say their child is growing too fast and beg that time slow down. For a NICU parent, time is not moving fast enough and we pray our child grows bigger and stronger each day. Being present is a skill the NICU experience teaches that some parents treasure after years have passed. Families can learn to slow down and be present in the moment.

From LaToshia’s experience: During our NICU stay, we were blessed that our children survived; however, there may be feelings of despair, grief, and sadness that are felt as well. I have experienced the pain of not being sure my son would survive in one second and then the joy of him squeezing my finger the next. There are major and mini blessings along the journey.

Having a child in the NICU takes away time from daily activities with other family members. It is a financial strain on families with the added expenses of eating out or eating convenience foods as well as the transportation cost for NICU visits. Families must take the time to learn about the staff and the schedules, bond with their baby, and learn about the medical care being received. This is just the tip of the iceberg. Families must juggle their everyday lives outside of the unit while the balance of these two worlds is just out of reach. Having peer support is a cornerstone of support that is not easily accessible for families. Many desire to connect with families for guidance. *Some NICUs organize opportunities for families to attend monthly dinners. These social supports give families an outlet, reduce feelings of isolation, and build community.*

Due to many factors, families of color find it increasingly more difficult to gather resources and supports to help their families thrive [10,11]. The inequities experienced highlight the need to address the social determinants of health and barriers in the healthcare system [12]. Black infants have twice the incidence of infant mortality and preterm birth compared to other US racial/ethnic groups [13,14]. From LaToshia’s perspective, her micro preemie triplets were born in a geographic location where her babies were 6 times more likely to die compared to babies of a different race. Implicit bias, racism, and the lack of diversity in the medical profession are some added barriers to thriving in the NICU for families of color [15]. Connecting with the care team and building a shared understanding is key to survival. For families of color, questions like, will they understand my baby’s needs? Can I share my concerns without retribution? Will they look down on me? Will they see my advocacy as love for my baby? Will they give my baby the best care? Will I be seen and heard? Will I have autonomy as a parent in the NICU like other parents? This is another layer of trauma that is not discussed enough in NICU experiences. Feelings of discomfort in a new environment and not having a place to voice your concerns, can cause additional care team dysfunction and impact the health of the child. *A collaborative care model must be established to leverage the parents’ perspective. Parents observe and process all communications with medical staff. Their child is their sole focus and only patient; compared to medical staff who see dozens of patients, parents are the only constant caregiver post discharge [16].*

From LaToshia’s experience: There were several times when I caught things that the staff missed about my babies. I was able to see the trend of how removing hydrocortisone caused a downturn for baby C and once the doctor mentioned removing it during rounds, I asked why. I explained my reason and he confirmed my observation via the medical records. We kept my son on the medication uninterrupted for another week to allow more time for his fragile body to catch up. This was a game-changer for his progress. If I had not developed a relationship with the provider to raise my concerns, I do not think my son would be here.

From Jessica’s experience: I found it critically important to ask questions and be involved in medical decision making. At one point the medical team recommended my son get a tracheostomy. When I asked why, they said “so that he can go home sooner”. This is when I realized that the hospital’s priorities and my family’s priorities may not be aligned. I did not want my son to come home as soon as possible with extra medical equipment and additional long-term care needs. I wanted my son to come home when he was medically stable and stronger. I pushed back and through an unexpected turn of events he was on life support a week later. He may not have survived a highly invasive medical procedure if we had proceeded with the tracheostomy.

Building trust is key to ensuring families feel supported. Who communicates information about services and programs matters since families’ experiences may have not been positive with similar services in the past [17]. Families must understand their options in terms of programs and services. Care team members need to approach families in a trauma-informed manner with an equity lens [18]. NICUs can shift their culture to support families by incorporating educational opportunities to raise awareness and deepen understanding of the lived experience, take time to listen with empathy to build trust, address systemic issues like the lack of coordinated support available for families, and partner with other units to find out best practices to improve the culture of care in the NICU [19]. Equity cannot be accomplished alone. Partnerships and collaboration are key to increasing equity for families.

A social worker, care coordinator, or patient navigator can truly make a difference in reducing anxiety and burden on the family. These professionals can assist in navigating financial support and insurance options and providing referrals that are key to health equity [20]. They can answer questions, coordinate appointments, identify future service needs, and help the family plan accordingly. Many NICUs struggle with having enough social workers to support families. Lactation consultants are important supports to discuss short- and long-term feeding goals. Given the disparities in breastfeeding rates for moms of color, it is also important to have lactation support that represents the population served to reduce further trauma around breastfeeding due to the history of women of color in our society [21]. NICU bedside psych/trauma support can help with emotional healing. Some families may even benefit from post-traumatic stress disorder (PTSD) treatment. *There are opportunities to care for the dyad of mom and baby while in the NICU. Mothers often prioritize the health of the baby while neglecting their own health needs. Caring for both mom and baby can have an impact on the baby. Both mothers and fathers may experience postpartum depression [22,23]. Targeted supports can make the experience less traumatic for the family and more tolerable.*

The support of postpartum doulas can shift the NICU culture. A postpartum doula is knowledgeable about the recovery process for the mom, lactation, and baby care. A curriculum that guides the education and NICU support efforts can establish a standard for care. Doulas can provide evidence-based information, link to local supports, hold community events, provide emotional support to NICU families, and transitional support at discharge while the family navigates their new normal back in their community. *Doula support can be tied into the NICU follow-up visits to fill a gap in care to better support families.*

Medical providers can help overcome barriers to families obtaining support services in the NICU. Federal and private funding can support additional staffing and programming. Peer support opportunities can be offered at various timepoints to connect families with similar experiences. *Doctors and nurses can also ask families on advisory councils about supportive language and how best to communicate with families in the NICU.*

## 7. Discussion

We drew on our knowledge of life course principles to identify concrete recommendations that could lead to actionable interventions to influence the health trajectory of families and children over time. Twelve key features were identified by the Health Resources and Services Administration, Maternal and Child Health Bureau’s Life Course Intervention Research Network, consisting of over 75 professionals from various fields.

These interventions aim to:

1. Optimize health trajectories;
2. Focus on developmental stages;
3. Have a longitudinal emphasis;
4. Be timed strategically;
5. Address multiple layers of the environment where children grow;
6. Integrate multiple dimensions for a proactive health system;
7. Enhance emerging health development capabilities;
8. Be co-designed by diverse research teams and stakeholders;
9. Center around families;
10. Build on strengths of families;
11. Adopt anti-racist strategies;
12. Prioritize health equity.

Our recommendations aligned with the list of life course intervention characteristics is meant to initiate broader discussions and cultivate change (Table 1). Integrating these features into interventions can potentially enhance health trajectories, rectify crucial developmental processes, and mitigate negative outcomes [3].

## 8. Conclusions

Caring for a medically complex child comes with its challenges. Children with special health care needs are more severely impacted by the adverse effects of inequities and the social determinants of health [26]. Recognizing the toll the NICU experience can have on the family and identifying the appropriate support can change the culture of the NICU. From our personal experiences, we believe the recommendations described can transform NICU care and improve outcomes for both families and babies.

## Figures and Tables

**Table 1:** Recommendations aligned with life course intervention characteristics.

Recommendation	Life Course Intervention Characteristic	Definition
Develop an advisory council with parents to address the holistic needs of families	Co-design	Designed by stakeholders (individuals, families, communities) and professionals working together.
	Family Centered	Recognize and support the unique role of families as incubators of early health development, with potential to build family resilience and buffer children from adverse experiences.
	Multi-level or Holistic	Designed to improve more than one aspect of the ecosystem in which children are born, live, learn, and grow, considering social and cultural context.
If there is a high likelihood for a NICU stay, provide families with supports as early as pregnancy ○ What to expect during a NICU stay ○ What are the expectations of parents while in the NICU ○ Sort out logistics of NICU visits ○ Discuss work schedule accommodations ○ Learn visitation policies ○ Provide psychological support to cope ○ Provide educational opportunities ○ Educate families on the importance of shared decision making so that they understand their role as a partner early on	Strategically Timed Family Centered	Target a critical or sensitive period of development, or a transition or turning point, to intervene with maximum efficacy and impact. Timing is multidimensional, including duration and frequency of intervention as well as stage of the life course.
	Addresses Emerging Health Capacities	Designed to support and enable processes leading to the development of capacities for positive health not just management or prevention of disease.
Build an interdisciplinary network of supports that carry parents through the NICU experience and beyond	Vertically, Horizontally and Longitudinally Integrated	Aimed at integrating services, programs, and other protective factors, including those outside the medical care sector, at all levels, in order to create a seamless, forward-leaning, health-optimizing system.
Give families opportunities to bond with their babies with supports as needed	Developmentally FocusedFamily Centered	Grounded in the knowledge that health development takes place from preconception through adulthood, and that each stage affects health development in subsequent stages. Strongly process oriented.
Discuss developmentally appropriate interactions with families and describe the cues that may indicate overstimulation [24]	Developmentally Focused	Grounded in the knowledge that health development takes place from preconception through adulthood, and that each stage affects health development in subsequent stages. Strongly process oriented.
Create a nurturing nursery-like environment in the NICU	Multi-level or Holistic	Designed to improve more than one aspect of the ecosystem in which children are born, live, learn, and grow, considering social and cultural context.
Teach a positive, growth mindset	Optimization Focused	Aimed at optimizing health trajectories rather than simply preventing or treating specific health problems.
	Addresses Emerging Health Capacities	Designed to support and enable processes leading to the development of capacities for positive health not just management or prevention of disease.
	Longitudinally Focused	Aimed at improving health reserves and resilience in early life that will contribute to disease prevention later in life.
Focus on progress and give hope when possible	Optimization Focused Strengths based	Builds on child, youth, family, and community strengths to build health reserves and to create adaptations to circumvent challenges.
Encourage families to make connections with other NICU families; facilitated support groups or ad hoc conversations	Family Centered	Recognize and support the unique role of families as incubators of early health development, with potential to build family resilience and buffer children from adverse experiences.
Empower families to be an advocate for their child	Family Centered	Recognize and support the unique role of families as incubators of early health development, with potential to build family resilience and buffer children from adverse experiences.
Build trust by establishing a collaborative model for care	Collaboratively co-designed	Designed by stakeholders (individuals, families, communities) and professionals working together.
Provide medical updates in plain language that is easy to understand	Family Centered	Recognize and support the unique role of families as incubators of early health development, with potential to build family resilience and buffer children from adverse experiences.
Learn about each family’s lived experience and be sensitive to each circumstance	Family Centered	Recognize and support the unique role of families as incubators of early health development, with potential to build family resilience and buffer children from adverse experiences.
Schedule regular Diversity, Equity, Inclusion and Belonging (DEIB) training for staff [25]	Health Equity Focused	Interventions support health equity recognizing that different circumstances and contexts warrant different intensities of intervention. Interventions are designed to help the most disadvantaged.
	Anti-racist	Interventions incorporate anti-racist principles and consider the potential role of, and effective responses to, racism.
Connect to community-based organizations to provide social supports and resources for families	Vertically, Horizontally and Longitudinally Integrated	Aimed at integrating services, programs, and other protective factors, including those outside the medical care sector, at all levels, in order to create a seamless, forward-leaning, health-optimizing system.
Screen parents for anxiety and depression	Strategically Timed	Target a critical or sensitive period of development, or a transition or turning point, to intervene with maximum efficacy and impact. Timing is multidimensional, including duration and frequency of intervention as well as stage of the life course.
Hire social workers, care coordinators, patient navigators, postpartum doulas, psychologists, and psychiatrists in the NICU to support parents	Vertically, Horizontally and Longitudinally Integrated	Aimed at integrating services, programs, and other protective factors, including those outside the medical care sector, at all levels, in order to create a seamless, forward-leaning, health-optimizing system.

Authors authorized use [1].

## Data Availability

Not applicable.
